# Factors Influencing Cancer Risk Perception in High Risk Populations: A Systematic Review

**DOI:** 10.1186/1897-4287-9-2

**Published:** 2011-05-19

**Authors:** Jon C Tilburt, Katherine M James, Pamela S Sinicrope, David T Eton, Brian A Costello, Jantey Carey, Melanie A Lane, Shawna L Ehlers, Patricia J Erwin, Katherine E Nowakowski, Mohammad H Murad

**Affiliations:** 1 Division of General Internal Medicine, Mayo Clinic, Rochester, Minnesota, USA; 2 Biomedical Ethics Research, Mayo Clinic, Rochester, Minnesota, USA; 3 Knowledge and Encounter Research Unit, Mayo Clinic, Rochester, Minnesota, USA; 4 Department of Psychiatry & Psychology, Mayo Clinic, Rochester, Minnesota, USA; 5 Department of Oncology, Mayo Clinic, Rochester, Minnesota, USA; 6 Division of Health Care Policy & Research, Department of Health Sciences Research, Mayo Clinic, Rochester, Minnesota, USA; 7 Mayo Clinic College of Medicine, Mayo Medical Library, Mayo Clinic, Rochester, Minnesota, USA; 8 Division of Preventive Medicine, Mayo Clinic, Rochester, Minnesota, USA

## Abstract

**Background:**

Patients at higher than average risk of heritable cancer may process risk information differently than the general population. However, little is known about clinical, demographic, or psychosocial predictors that may impact risk perception in these groups. The objective of this study was to characterize factors associated with perceived risk of developing cancer in groups at high risk for cancer based on genetics or family history.

**Methods:**

We searched Ovid MEDLINE, Ovid Embase, Ovid PsycInfo, and Scopus from inception through April 2009 for English-language, original investigations in humans using core concepts of "risk" and "cancer." We abstracted key information and then further restricted articles dealing with perceived risk of developing cancer due to inherited risk.

**Results:**

Of 1028 titles identified, 53 articles met our criteria. Most (92%) used an observational design and focused on women (70%) with a family history of or contemplating genetic testing for breast cancer. Of the 53 studies, 36 focused on patients who had not had genetic testing for cancer risk, 17 included studies of patients who had undergone genetic testing for cancer risk. Family history of cancer, previous prophylactic tests and treatments, and younger age were associated with cancer risk perception. In addition, beliefs about the preventability and severity of cancer, personality factors such as "monitoring" personality, the ability to process numerical information, as well as distress/worry also were associated with cancer risk perception. Few studies addressed non-breast cancer or risk perception in specific demographic groups (e.g. elderly or minority groups) and few employed theory-driven analytic strategies to decipher interrelationships of factors.

**Conclusions:**

Several factors influence cancer risk perception in patients at elevated risk for cancer. The science of characterizing and improving risk perception in cancer for high risk groups, although evolving, is still relatively undeveloped in several key topic areas including cancers other than breast and in specific populations. Future rigorous risk perception research using experimental designs and focused on cancers other than breast would advance the field.

## Background

*Perceived risk *is an important subjective psychological phenomenon related to threat appraisal that is closely intertwined with judgments about susceptibility to disease as well as the probability of benefit from interventions [1]. It remains an integral component of several theories of health behavior (e.g. the Health Belief Model, the Precaution Adoption Model, or the Transactional Model of Stress and Coping) [2]. Thus, risk perception is an essential component of health behavior in cancer generally, and in hereditary cancers in particular.

Compared to cancer risk perception in the general population, experiencing a close family member going through treatment for cancer or having a known genetic susceptibility to cancer has life-altering implications, including how one processes risk information [3]. At-risk family members or those with known mutations may have to make important decisions based upon their risk perceptions, including whether to undergo prophylactic surgery or subsequent genetic testing, whether to disclose test results to family members, or whether to participate in experimental cancer screening (e.g. spiral computed tomography). Moreover, misperception of risk has been shown to both increase and decrease use of preventive health services and therefore can have significant implications for the health of those at greater than average risk of developing cancer.

The existing research on cancer risk perception is scattered across disciplines (i.e. health services research, psychooncology, health communication) and across populations (i.e. cancer patients and the general public) [2]. In a recent narrative review, Klein and Stefanek discuss the role of *innumeracy, heuristics, motivational factors*, and *emotional influences *in shaping risk perception and the implications for cancer risk perception [4]. They conclude that the psychology of risk perception should elicit caution among clinicians hoping to accurately convey risk information to patients and call for a research strategy that spans the fields of medical decision-making and health communication.

The little remaining empirical literature synthesizing data regarding cancer risk perception either focuses on a specific cancer such as breast cancer [5], or describes interventions to improve risk communication in cancer. However, little is said about the key clinical, demographic, or psychosocial predictors that may shape risk perception [6], all of which are critical factors for tailoring interventions that align patients' perceptions of risk with their calculated risk [7].

We undertook a systematic review to rigorously characterize the existing empirical literature on factors that may influence perceived risk of getting cancer for those at high risk for cancer in order to form an empirically-grounded conceptual model for future cancer risk communication research.

## Methods

The report of this protocol-driven systematic review adheres to the Preferred Reporting Items for Systematic reviews and Meta-Analyses (PRISMA) statement [8]. Protocol details are available upon request.

### Eligibility criteria

Studies were eligible if they evaluated associations between psychosocial, clinical, or demographic factors and cancer risk perception (or similar terms such as perceived susceptibility or risk interpretation). Eligible studies sampled individuals who had one or more known or suspected non-modifiable risks for hereditary cancer such as family history or having a positive genetic test. We excluded studies that sampled exclusively from the general population, average risk populations, or healthcare providers. We initially included studies regardless of their design or sample size and excluded review articles, commentaries, letters not containing original data, and studies that were exclusively qualitative due to the unfeasibility of data abstraction. We excluded studies in which the focus was on establishing associations of risk factors with outcomes or behaviors if they did not evaluate predictors of risk perception, interpretation or communication. Similarly, we excluded educational interventions aimed exclusively at raising awareness of risk. The characteristics of reviewed studies are provided in Table 1.

**Table 1:** Description of 53 studies reporting clinical, demographic or psychosocial factors related to risk perception in cancer in patients at high risk for cancer

Study Characteristics	No. (%) of Studies
**Study Design**	
Experimental (e.g. RCT)	4 (8)
Observational	49 (92)
Cross-sectional	20 (38)
Cohort (retrospective or prospective)	15 (28)
Case-control	4 (8)
Other	10 (19)
**Study Population Type^a^**	
Non-Genetic	36 (68)
Genetic	17 (32)
**Study Population Characteristics**	
Gender Representation	
Male only	2 (4)
Female only	37 (70)
Mixed	14 (26)
Hispanic ethnicity reported	6 (11)
Race reported^b^	36 (67)
White/Caucasian	29 (55)
Black/African American	11 (21)
Asian	6 (11)
American Indian	4 (8)
Hawaiian/ Pacific Islander	3 (6)
Other	17 (31)
**Type(s) of Cancer Studied**	
Breast	34 (64)
Ovarian	16 (30)
Colon	12 (23)
Prostate	2 (4)
**Utilized a Theoretical Model of Health Behavior**	7 (13)
**Methods of Measuring Risk Perceptions^c^**	
Single Item	37 (70)
Multiple Item	15 (28)
Categorical Measures	35 (66)
Absolute	18 (34)
Comparative	23 (43)
Continuous	25 (47)
Absolute	24 (45)
Comparative	3 (6)
**Factors**	
Clinical	39 (74)
Demographic	16 (30)
Psychosocial	32 (60)

^a ^Some studies included a mix of high risk and average risk/healthy individuals.

^b ^Some studies included multiple race categories, therefore percentages do not add to 100.

^c ^Some studies included multiple measures, therefore percentages do not add to 100.

### Search strategy

An expert reference librarian designed and conducted an electronic search strategy with input from study investigators focused on factors that may influence risk perception relevant to clinical care. We searched Ovid MEDLINE, Ovid Embase, Ovid PsycInfo, and Scopus from inception through April 2009. Only English language articles were selected. The core concepts were risk (MeSH term: risk management) and cancer (MeSH terms: neoplasms limited to diagnosis, treatment, epidemiology prognosis, mortality). A series of terms and text words were added such as patient education, attitude of patients and health care personnel, educational status, comprehension, communication in the context of decision making, choice, preferences, and uncertainty. In addition, we sought additional references from bibliographies of eligible studies and content experts. A detailed list of subject headings and text words is available upon request.

### Assessment of study eligibility

Teams of two reviewers working independently and in duplicate screened all abstracts and titles and, upon retrieval of candidate studies, reviewed the full text to determine eligibility. Disagreements were resolved through discussion or by arbitration through a third reviewer. The mean chance-adjusted agreement (kappa) was 0.70.

### Data extraction and synthesis

Teams used standardized forms to extract descriptive, methodological, and key variable data from all eligible studies. We used an online reference management system for systematic reviews to conduct study selection and data extraction (SRS 4.0 Mobius Analytics, Ottawa, Ontario, Canada).

Data collected from the vetted studies included study design, description of the population, description of the risk of cancer, analytical techniques, and the theoretical model tested. From each study, we extracted data regarding the type and strength of associations between psychosocial, clinical, and/or demographic factors and cancer risk perception, interpretation and/or communication. Disagreements between reviewers at this stage were also resolved by discussion or third-reviewer arbitration.

**Figure 1:**
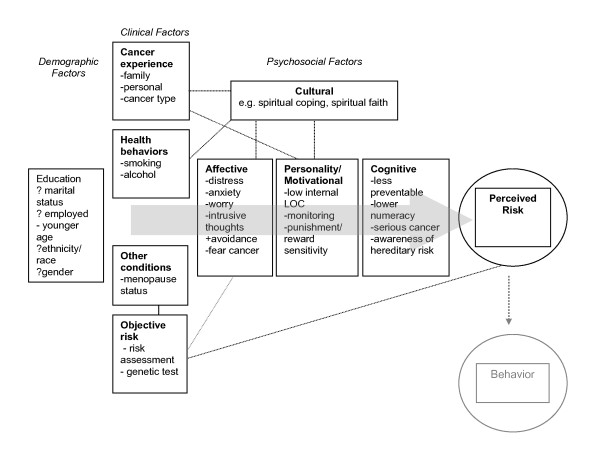
**Interrelated factors associated with cancer risk perception**. Conceptual model of factors thought to be associated with perceived risk for cancer.

Considering the heterogeneity of the included studies in terms of design, population, type of cancer and outcome measures, and because the review was designed to generate (not test) hypotheses for future research, we did not conduct a meta-analysis. Data were tabulated and categorized according to the factors that affected risk perception. Narrative synopses describing the tested associations and the main findings of each study are presented (Tables 2 &3). These summaries provided the empirical basis upon which to build a conceptual model of factors influencing risk perception in cancer (Figure 1). Since the included studies were mostly of a cross-sectional design, the quality of evidence was considered to be low and at high risk of bias. Therefore we did not extract data about bias protection measures in the included studies.

**Table 2:** Characteristics of 36 studies reporting clinical, demographic, and/or psychosocial factors related to cancer risk perception in high risk populations not related to genetic susceptibility testing

First author, year	Design	Cancer Type	No. sub-jects	Age (years)	Gender M/F/ M+F	Tested Factors Influencing Risk Perception			Study Synopsis
							
						Clinical	Demographic	Psychosocial	
Haas, 2005 [30]	Observational, prospective cohort	Breast	1619	Range 40-74	F	Previous childbirth; + FH of breast cancer; *BMI; Prior breast biopsy; Prior abnormal mammogram*	Age; Race; *Marital status; Education level*		Studied women's objective & subjective risks for developing BC. Younger women overestimated future BC risk. For women at average BC risk, Asian Pacific Islanders and women with FH of BC were more likely to overestimate risk. For women at high BC risk, younger women were more likely to accurately perceive risk, and black women (vs. whites) were less likely to accurately perceive risk.

Rowe, 2005 [31]	Observational, cross-sectional	Breast	66	Mean 40, Range 25-59	F	+ FH of breast cancer	Marital status; *Age; Ethnicity; Employment status*	Locus of Control; Breast cancer-specific control	Studied women with & without FH of BC. Married women more likely to perceive lower risk of BC than unmarried women. Women with +FH of BC perceived higher risk for BC. Internal locus of control and breast cancer-specific control were significantly related to women's perceived likelihood of remaining free of breast cancer.

Gil, 2003 [47]	Observational, case-control	Breast	84	Range 18-53	F	+ FH of breast cancer			Studied distress, perception of BC risk, screening behaviors, coping skills, personality and quality of life in Spanish cohort of women with & without FH of BC. Women with FHBC overestimated their risk of developing breast cancer.

Lebel, 2003 [34]	Observational, cross-sectional	Breast	25	Mean 56	F	*+ FH of breast cancer*		Distress; Venting & denial coping strategies	Interviewed women with suspicious mammograms at two time points: immediately after being put on biopsy wait-list and immediately before biopsy. Higher perceived risk of malignancy correlated with distress and use of venting and denial coping strategies.

Fang, 2003 [48]	Observational, cross-sectional	Ovarian	76	Mean 42, Range 22-71	F	*+ FH of breast or ovarian cancer*			Studied women with FH of ovarian cancer and their intention to undergo prophylactic oophorectomy. Perceived risk levels were not associated with family history of ovarian cancer or with family history of breast or ovarian cancer.

Hatcher, 2001 [26]	Observational, prospective cohort	Breast	143	Grp 1 median 38, Grp 2 median 40	F	Prophylactic mastectomy status			Studied women with increased risk of developing BC who were offered bilateral prophylactic mastectomy and who accepted or declined the surgery. Acceptors were more likely than decliners to believe it inevitable that they would develop breast cancer.

Wellisch, 2001 [49]	Observational, prospective cohort	Breast	430	Mean 43, Range 15-78	F	Depression status			Studied women who presented to a high risk breast cancer clinic. When estimating their own risk of developing breast cancer, women scoring above the CES-D (depression scale) cut-off point reported higher personal risk estimates than did women scoring below the cut-off point.

Audrain, 1997 [35]	Observational, prospective cohort	Breast; Ovarian	256	Mean 44, Range 21-73	F			General distress; Perceived control over BC	Studied women with a family history of breast or ovarian cancer who self-referred for genetic counseling. Women with higher levels of general distress had heightened BC PR, though this effect was moderated by having low perceptions of control over the development of breast cancer.

Schwartz, 1995 [43]	Observational, cross-sectional	Ovarian	103	Mean 42, Range 18-74	F	Age of diagnosis for FDR with ovarian cancer		Intrusive thoughts; Attentional Style; *Mood disturbance*	Studied women with ≥1 FDR with ovarian cancer. Perceived risk of developing ovarian cancer was positively correlated with intrusive thoughts and monitoring, and was negatively correlated with the age of diagnosis for FDR relative with ovarian cancer.

Zikmund-Fisher, 2008 [50]	Experimental	Endometrial	631	Mean 59, Range 40-74	F			Numeracy	Studied women with elevated BC risk. Higher numeracy was significantly associated with lower perceived risk of side-effects of tamoxifen, including endometrial cancer.

Mellon, 2008 [3]	Observational, familial dyads	Breast; Ovarian	292	Grp 1 mean 51, Grp 2 mean 41	F	Cancer type of affected relative; +FH of cancer	Race; Age; Income	Cancer worry	Studied dyads of adult breast & ovarian cancer survivors and their unaffected female relatives. Caucasian race was associated with higher risk perceptions, as was income, older age, family history of cancer, cancer type, and high levels of cancer worry.

Salsman, 2004 [13]	Observational, cross-sectional	Ovarian	624	Grp 1 mean 57, Grp 2 mean 57	F	*Ovarian cancer screening status*			Studied women undergoing routine transvaginal sonography screening for ovarian cancer and an age and education-matched healthy comparison group. Perceptions of lifetime risk for OC did not differ between the two groups.

Beebe-Dimmer, 2004 [40]	Observational, cross-sectional	Prostate	111	Mean 54, Range 33-78	M	+ FH of prostate cancer	Age; *Marital status; Education Level*	Concern	Studied men whose brothers had been diagnosed with prostate cancer. Men younger than their affected brother, those with more than one affected FDR, and those with higher levels of concern had higher estimates of personal risk for prostate cancer.

Lobb, 2004 [22]	Observational	Breast	158	Grp 1 mean 39, Grp 2 mean 51	F	Receiving written summary of genetic counseling session			Studied women from high risk BC families to assess how communication regarding genetic testing for BC was associated with various features of communication. They found that having received a written summary of the results was associated with more accurate risk perception.

Andrykowski, 2002 [36]	Observational, case-control	Breast	176	Grp 1 mean 44, Grp 2 mean 45	F	*Undergoing breast biopsy*		Impact of Events Scale-intrusion & avoidance	Studied women with benign breast biopsy and a healthy comparison group. No differences were found between groups in perceived risk of BC. Perceived BC risk was significantly negatively associated with intrusion and avoidance scores on the Impact of Events Scale.

Royak-Schaler, 2002 [32]	Observational, cross-sectional	Breast	141	Range 23-81	F	*> 1 relative with cancer*	*Race*	Having more complete discussion with doctor	Studied FDRs of breast cancer patients. Provider discussions about FH and personal risk were accompanied by increases in risk perception and promoted compliance with screening goals.

Elit, 2001 [25]	Observational, cross-sectional	Ovarian	40	Mean 55	F	Oophor-ectomy status			Studied women with FH of OC who had undergone prophylactic oophorectomy. Perceived risk for OC was found to decrease significantly after surgery.

Vernon, 2001 [27]	Observational	Colon	1955	No means given	M	+FH of polyps or colon cancer; Colon screening exam status	Age; Education Level	Degree of familial support; Cancer worry	Studied male autoworkers who participated in a trial to increase CRC screening. At baseline, a positive association was found between PR of cancer and positive FH, family support for screening, and worry about being diagnosed.

Collins, 2000 [39]	Observational	Colon	127	Mean 47	M/F			Cancer worry	Studied patients presenting to a familial CRC clinic. A significant negative association was found between PR of bowel cancer and cancer worry.

Erblich, 2000 [38]	Observational, cross-sectional	Breast	148	Mean 42	F	*Maternal death due to BC; Serving as caregiver for mother with BC*		Anxiety; IES-intrusion & avoidance; General distress; *BSI depression*	Studied women with and w/out FDRs with BC. Among women with FH of BC, perceived risk was positively correlated with anxiety, intrusion & avoidance thoughts on the Impact of Events Scale, and global distress.

Glanz, 1999 [16]	Observational, cross-sectional	Colon	426	Mean 50, Range 19-84	M/F		Education Level	Awareness of CRC family history	Studied FDRs of patients with CRC. Being a college graduate and having an awareness of a relative with CRC cancer were independently and positively associated with risk perception.

Zakowski, 1997 [19]	Observational	Breast	89	Mean 42, Range 23-55	F	Objective cancer risk; +FH of breast cancer; Death of parent to cancer	*Age at time of parent(s)' death*	IES-intrusion & avoidance	Studied women with and without FH of BC. Higher PR of BC was found in women with FH of BC and women whose parent(s) had died of cancer. Results suggested that high PR predicts high levels of intrusive thoughts and avoidance regarding BC.

Stefanek, 1995 [51]	Observational	Breast	164	Grp 1 mean 37, Grp 2 mean 38	F	Prophylactic Mastectomy status			Studied women with ≥1 FDR diagnosed with BC who underwent prophylactic mastectomy, expressed an interested in surgery, or did not express an interest. Women who underwent surgery had significantly higher perceived risk than women in the non-interest group.

Lerman, 1994 [28]	Observational, cross-sectional	Breast	Grp 1 n = 179, Grp 2 n = 238, Grp 3 n = 363	Grp 1 range 30-75, Grp 2 range 20-75, Grp 3 range 20+	F		Age		Studied women with a FH of BC presenting to three different clinics. At one site, women in the 30-34 and 50+ categories were significantly less likely to perceive themselves as having and elevated risk than were women in other age groups. No other significant differences by age were found in the two other study sites.

Bondy, 1992 [52]	Observational, cross-sectional	Breast	30604	Grp 1: 61% over age 60, Grp 2: 51% under age 50	F	Objective risk based on Gail model; Degree of FH			Studied women with and w/out FDRs affected by BC. Women with the highest relative risk scores for breast cancer (based on the Gail model) more likely to perceive high lifetime risk of breast cancer compared to women in lower risk categories. Women with FDRs affected by breast cancer had higher perceived risk, particularly when those relatives were their mother and sister.

Blalock, 1990 [14]	Observational, cross-sectional	Colon	295	Grp 1 mean 56, Grp 2 mean 59	M/F		Race	Self-perceived heredity	Studied people with CRC-affected siblings and an average risk comparison group. High risk individuals were more likely to rate heredity as a risk-increasing factor than as a risk-decreasing factor, and whites in the high risk group were more likely than blacks to rate heredity as a risk-increasing factor.

Watson, 1999 [23]	Observational, prospective cohort	Breast	282	Median 37, Range 19-76	F	Having undergone genetic counseling		Intrusive thoughts; Cancer worry	Studied women with a FH of BC. Genetic counseling produced a modest shift in the accuracy of perceived lifetime risk of BC. Women with a higher than average PR of BC were more likely to report intrusive thoughts and cancer worry.

Cunningham, 1998 [37]	Observational, case-control	Breast	132	Grp 1 mean 50; Grp 2 mean 49	F			Cancer worry	Studied women with benign breast problems and a healthy comparison group. BC risk perceptions were found to mediate differences between the BBP and healthy comparison group in breast cancer worry.

Miller, 2005 [42]	Observational, prospective cohort	Breast; Ovarian	279	Mean 46	F			Monitor status	Studied women who expressed concerns about their risk for BC or OC during self-initiated calls to a Cancer Information Service. High monitors, who typically attend to and seek information, demonstrated greater increases in knowledge and perceived risk over the 6-month interval than low monitors.

Emery, 2007 [21]	Experimental	Breast; Colon; Ovarian	246	-	M/F	Referral to a genetics clinic			Studied patients referred to the Regional Genetics Clinic by practices randomized to use either Genetic Risk Assessment on the Internet with Decision Support (GRAIDS) software or current best practices. Patients who were not referred from GRAIDS practices to the genetics clinic showed lower mean risk perception than those who were referred.

Bjorvatn, 2007 [20]	Observational, cross-sectional	General Cancer Risk	213	Mean 42, Range 18-80	M/F	Undergoing genetic counseling		Cancer worry	Studied patients from genetic outpatient clinics of three Norway hospitals. Perceptions of risk were significantly reduced and more likely to be accurate after genetic counseling compared to before. After counseling, higher PR of developing cancer was found to be correlated with higher worry.

Quillin, 2006 [45]	Observational, cross-sectional	Breast	899	Mean 50, SD 8	F	+ FH of cancer	Race; *Education*	Spiritual coping	Studied women in the Women Improving Screening Through Education & Risk Assessment (WISER) study. Higher levels of spiritual coping were associated with a lower perception of BC risk, but only for women with a self-reported FH of cancer. African-Americans were more likely to perceive lower risk of BC than Caucasians.

Lipkus, 2006 [53]	Experimental	Colon	160	Grp 1 mean 56; Grp 2 mean 55; Grp 3 mean 58; Grp 4 mean 56	M/F	Possession of colorectal cancer risk factors		Exposure to different types risk communi-cation; *ambivalence to screening*	Studied adults who were off-schedule for having a fecal occult blood test. Participants who thought they had more CRC risk factors reported greater perceived absolute and comparative risk.

Cameron, 2006 [54]	Observational, cross-sectional	Breast	303	Range 18-82; Grp 1 mean 44; Grp 2 mean 43; Grp 3 mean 25	F	+FH of breast cancer		Worry	Studied general practitioner clinic attenders, university students, and FDRs of BC survivors. A moderate correlation between perceived risk and worry was found. FDRs of BC survivors reported higher perceived risk than university students and clinic attenders.

Madalinska, 2005 [55]	Observational, cross-sectional	Breast	846	Grp 1 mean 49; Grp 2 mean 47	F	OC preventive measures			Studied women at high risk of OC. PR of developing BC was significantly lower among women who had undergone prophylactic bilateral salpingo-oophorectomy than women undergoing gynecologic screening.

Cappelli, 2005 [10]	Observational, case-control	Breast	110	Mean 16	F	Family risk status			Studied pairs of adolescent daughters whose mothers had been treated for BC and daughters of healthy mothers. Compared to adolescent daughters of parents with no serious illnesses, daughters of mothers with BC reported elevated perceived risk of developing BC and an elevated risk of having a BRCA mutation.

NOTE: Factors in italicized text indicate non-significant associations. Common abbreviations include PR = perceived risk; FH = family history; BC = breast cancer; CRC = colorectal cancer; OC = ovarian cancer; FDR = first-degree relative.

**Table 3:** Characteristics of 17 studies reporting clinical, demographic, and/or psychosocial factors related to cancer risk perception in patients with established genetic cancer susceptibility

First author, year	Design	Cancer Type	No. sub-jects	Age (years)	Gender M/F/ M+F	Tested Factors Influencing Risk Perception			Study Synopsis
							
						Clinical	Demographic	Psychosocial	
Domanska, 2007 [29]	Observational, retrospective cohort	Colon, Endometrial	47	Mean 49, Range 24-76	M+F	*Personal history of cancer*	*Age;* *Sex*		Studied individuals with hereditary nonpolyposis colorectal cancer-causing mutations who underwent genetic counseling. Women and mutation carriers < 50 yrs reported highest PR for colon cancer. A personal history of HNPCC-related cancers was not associated with PR for colon cancer.

Cappelli, 2001 [9]	Observational	Breast; Ovarian	108	Grp 1 mean 40, Grp 2 mean 32	F	≥1 relative diagnosed with BC			Studied women with ≥1 relative diagnosed with BC and women from general population w/out cancer diagnosis. Women in high risk group had a higher overall perceived risk of getting cancer.

Peterson, 2008 [56]	Observational	General cancer risk; p53 muta-tion risk	92	Mean 50, Range 18-81	M/F			Cancer-specific distress	Studied individuals from Li-Fraumeni syndrome families at high risk of having a p53 mutation. Higher perceived risk of cancer and having a p53 mutation was associated with higher cancer-specific distress.

Codori, 2005 [33]	Observational, prospective cohort	Colon	101	Mean 44, Range 18-81	M/F	+FH of colorectal cancer; Objective risk of CRC; *Depressive symptoms*	Age; *Sex; Education Level*	Belief about preventability of CRC; Anxiety; *Coping Style; Tolerance for Ambiguity*	Studied adults with ≥ 1 relative diagnosed with CRC who received genetic counseling. Lower PR was associated with being older, having higher objectively estimated risk, having few or many FDRs with CRC, and beliefs about the preventability of CRC. A borderline association between PR and anxiety was also found.

Claes, 2004 [57]	Observational, prospective cohort	Colon; Endometrial	40	Grp 1 mean 41, Grp 2 mean 43	M/F			*Distress*	Studied patients who had a test for HNPCC. Perceived risk of CRC was not found to be associated with intrusion & avoidance measures in a distress scale.

Bruno, 2004 [58]	Observational, cross-sectional	Breast	677	Mean 45, range 23-78	F	+FH of breast or ovarian cancer			Studied women attending an outpatient cancer screening/prevention clinic in Italy. Only a minority perceived having a higher personal risk of BC than their peers, though this number was significantly higher in women with a FH of BC than those without one.

Van Dijk, 2003 [18]	Observational	Breast	241	< 30: 16% 30-39: 27% 40-49: 33% 50+: 25%	F	Objective risk; Having undergone genetic counseling			Studied women with personal or FH of BC and the impact of genetic counseling on perceived risk and worry. Undergoing genetic counseling resulted in more accurate perceptions of risk for breast cancer. Women with a higher PR for BC reported stronger intention to undergo prophylactic mastectomy.

Hensley, 2003 [59]	Observational	Ovarian	147	Median 47, Range 30-78	F	Menopausal status			Studied women at high risk for OC enrolling in a screening study. Premenopausal women were more likely than postmenopausal women to consider themselves at higher risk of ovarian cancer. When comparing themselves to others with similar family history, postmenopausal women considered themselves at higher risk for ovarian cancer.

Di Prospero, 2001 [24]	Observational	Breast; Ovarian	16	Mean 55, Range 39-83	M/F	Receipt of BRCA1/2 genetic test results			Studied individuals who received positive BRCA1/2 test results. Cancer risk perception increased after receipt of genetic test results.

Bratt, 2000 [41]	Observational, cross-sectional	Prostate	110	40-49: 35%; 50-59: 36%; 60-69: 27%; 70-72: 2%	M	Number affected family members/ deceased relatives		Cancer worry; Depression	Studied unaffected men with a pedigree consistent with hereditary prostate cancer. PR of cancer was positively correlated with both the number of prostate cancer-affected and deceased members in men's families. PR was also associated with symptoms of depression and cancer worry.

Codori, 1999 [17]	Observational, cross-sectional	Colon	258	Grp 1 median 44, Grp 2 median 50	M/F	Acceptance of genetic testing		Frequency of thoughts about CRC	Studied FDRs of patients with CRC. Those who accepted HNPCC testing had higher perceived risk compared to those who declined. The association between risk perception and uptake was dependent on frequency of cancer thoughts.

Rimes, 2006 [15]	Observational, prospective cohort	Breast; Colon; Ovarian	218	Mean 39, SD 10	M/F	+FH of cancer	Age	Anxiety	Studied people with a FH of colon or breast and/or ovarian cancer. Those with a FH of colon cancer had lower PR of developing cancer than people with a FH of BC and/or OC. Younger age predicted greater PR of developing cancer. Before receiving genetic counseling, higher anxiety was associated with higher PR of cancer.

Schwartz, 2000 [46]	Observational, prospective cohort	Breast; Ovarian	290	< 45 years: 31%	F	BRCA1/2 test 'uptake'		Spirituality/ Faith	Studied adult BC patients who had self-referred to the Cancer Assessment and Risk Evaluation Clinic at a cancer center. PR for BC and OC was found to be associated with patients' decision to undergo BRCA1/2 testing. This association was found to be modified by patients' degree of spirituality.

van Oostrom, 2007 [60]	Observational, prospective cohort	Breast; Colon; Ovari an	271	Grp 1 mean 43; Grp 2 mean 41	M/F	*Familial mutation type (HNPCC v. BRCA1/2)*; Mutation carrier status			Studied individuals undergoing genetic testing for a familial BRCA1/2 mutation or mutation predisposing to HNPCC. There were no differences between BRCA1/2 and HNPCC families in levels of perceived risk. For both groups, actual carriers reported greater perceived risk after disclosure of a positive test result.

O'Neill, 2006 [44]	Observational, prospective cohort	Breast; Ovarian	64	Mean 57, Range 36-80	F			Distress; Intolerance for uncertainty	Studied women with FH of BC who received uninformative BRCA1/2 results. 6 months after test result disclosure, patients who perceived an elevated BC risk and who difficulty coping with uncertainty reported high levels of ongoing distress.

Matloff, 2006 [12]	Experimental	Breast	48	Mean 49, Range 41-55	F	*Objective risk; Use of hormone therapy*		Cancer Worry	Studied menopausal women with ≥1 FDR with BC, some of whom received a personalized risk assessment intervention. Perceived risk and worry were significantly positively correlated at 6 mos follow-up.

Martin, 2006 [11]	Observational, retrospective cohort	Breast	56	Mean 44, Range 23-71	F	*Number of relatives with BC;*	*Age; Education Level*	*Depressive symptoms*	Studied women with a FH of BC. Age and education level were not found to be significantly associated with perceived risk of breast cancer. However; there was a slight trend toward a higher score on the depressive symptoms scale with a higher level of PR.

NOTE: Factors in italicized text indicate non-significant associations. Common abbreviations include PR = perceived risk; FH = family history; BC = breast cancer; CRC = colorectal cancer; OC = ovarian cancer; FDR = first-degree relative.

## Results

### Search results

**Figure 2:**
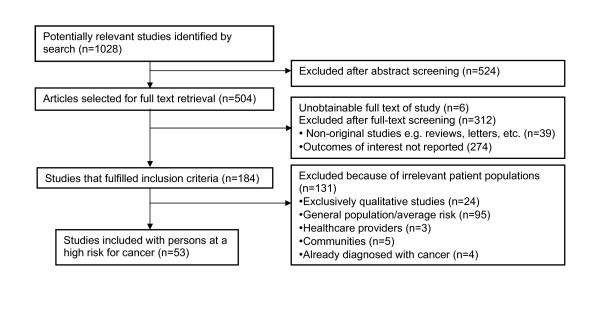
**Study selection process**. Flow diagram of how research studies were screened and selected.

Our search identified 1028 candidate articles for abstract review. After screening abstracts (when present), we excluded 524 articles and retrieved 504 full text articles. Of these, 184 fulfilled the basic inclusion criteria, but 131 of these were excluded for being exclusively qualitative, addressing risk perception among healthcare providers, including exclusively patients who already had cancer or patients with one or more modifiable risks for cancer, such as smoking. This left 53 articles that met all inclusion and exclusion criteria (Figure 2). Of those 53 studies, 36 reported participants with elevated risk who had not undergone genetic testing, and 17 reported participants who had undergone genetic testing.

#### Study designs

Table 1 describes the characteristics of the 53 included studies. Most of the studies (92%) had an observational study design and only four were experimental.

#### Study populations

Thirty-eight studies (70%) reported exclusively female populations, with most (14) of the remaining 16 including both genders. The majority (67%) included race information, but relatively few included non-white populations and made race-related inferences.

#### Cancers

The majority of studies (64%) addressed risk perception of breast cancer, followed by ovarian cancer (often along with breast cancer risk) (30%), colorectal cancer (23%), and prostate cancer (4%). Some studies examined multiple cancers.

#### Risk perception measures & theories

A range of self-reported measures of risk perception were employed across studies. These measures used a variety of total item numbers, categorical as well as continuous variables, and in some cases asked patients to estimate their absolute risk while in others participants were asked to compare their risk to a relevant comparison group (comparative risk). Thirty-seven studies used a single item measure of risk perception, 11 used two measures, three used three measures, and one study used four measures. Twenty-two studies assessed accuracy of risk perception. Of these, six were in studies where subjects had undergone some kind of genetic testing. We provide further details of risk perception measures in an accompanying appendix (see Additional file 1: Appendix).

Only 7 studies in our review referenced a specific theoretical model of health behavior as motivating or informing their research. These included the health belief model [9-13], the theory of planned behavior/theory of reasoned action [12,14], the cognitive behavioral model of health anxiety [15], and the precaution adoption model [16].

The major clinical, demographic, and psychosocial factors influencing risk perception in patient populations at high risk for cancer are described below. Tables 2 and 3 show results of the review stratified by risk perception in those who had not undergone genetic susceptibility testing as well as risk perception in those who had undergone such testing. Any differences between groups are discussed in the text.

### Clinical factors

A variety of clinical factors were associated with cancer risk perception. The most common association included a family history of cancer or precancerous lesions (n = 11). Others included type of cancer [3], objective cancer risk (e.g. from the Gail Model) [17-19], having received genetic counseling or written documentation of genetic testing results [20-23], and family history of death from the cancer of interest [19]. Clinical factors influencing risk perception among those who had undergone genetic susceptibility testing focussed primarily on the extent to which having undergone testing itself was a predictor of risk perception [17,18,24]. In several studies of patients with strong family history but no genetic testing, the retrospective observation of having undergone screening, prophylactic surgery, or biopsy was associated with risk perception [25-27].

### Demographic factors

#### Age

Ten studies examined associations between age and risk perception with variable findings. For instance, Rimes et al found that, among those with a family history of colon cancer, younger age predicted greater perceived risk of developing cancer [15]. Likewise, Lerman et al found that women in the 30-34 and 50+ categories were less likely to perceive themselves as having an elevated risk than were women in other age groups [28]. No studies tested the association between age and accuracy of risk perception.

#### Gender

Just two studies examined associations between gender and risk perception. Domanska noted that men and women differed in the proportions of those who perceived their lifetime risk of colorectal cancer as being greater than 60%, but these differences were not significant [29]. Since only 20% of all the studies focused on risk perception of non gender-specific cancers, there was minimal opportunity to evaluate the role of gender in cancer risk perception.

#### Race/Ethnicity

Only four studies explored the relationship between race/ethnicity and risk perception. Haas found that black women were less likely than white women to accurately perceive risk [30]; Mellon found that being Caucasian was associated with higher risk perception [3]; Blalock found that whites in a high risk group were more likely than blacks in the same group to rate heredity as a risk-increasing factor [14]. Others tested but did not find significant associations between race/ethnicity and risk perception [31,32].

#### Other Demographic Factors

Some studies also tested for associations between other demographic factors and cancer risk perception. Rowe tested but did not find any association between marital status, employment status and risk perception [31], while Glanz et al found that having a college education was directly associated with higher colorectal cancer risk perception [16].

### Psychosocial Factors

In addition to clinical and demographic factors that may influence risk perception, several key psychosocial factors were also identified. These include cognitive, affective, as well as personality and coping factors.

#### Cognitive

Beliefs about the nature of the participant's condition and its preventability were occasionally examined. Codori found that those who believed colorectal cancer was less preventable expressed a higher perceived risk of the disease [33]. Degree of awareness of one's family history or the hereditary nature of one's risk were also positively associated with risk perception [14,16].

#### Affective

Distress, anxiety and worry have been consistently associated with risk perception in multiple studies. Lebel found that overestimation of risk was associated with greater distress among those undergoing biopsy of breast lesions [34]. Audrain studied women with a family history of breast or ovarian cancer and found similar results [35].

Some studies (n = 3) examined the association between risk perception and existing measures of distress including the Impact of Events Scale (with the intrusion and avoidance subscales) [19,36,37], and the Total Mood Disturbance scale from the Profile of Mood States (POMS-TMD) [38]. Andrykowski showed a negative association between perceived risk and avoidance among women who had undergone biopsy [36]. That is, the greater the perceived risk, the less propensity for avoidance behavior. In contrast, Zakowski found that those whose parents had died of cancer had the highest levels of intrusive thoughts, avoidance and perceived risk [19].

Vernon's study of men at high risk of colorectal cancer [27] and Collins' study of patients presenting at a genetic testing clinic [39] showed that perceived risk was directly associated with worry about being diagnosed with colorectal cancer. Rimes, Beebe-Dimmer, and Bratt came to similar conclusions with analogous affective measures [15,40,41].

#### Personality and Coping

A few studies showed that the encoding pattern of "monitoring," (i.e., scanning for, attending to, and amplifying cancer threats) [42,43] as well as specific coping styles [33,44] were also correlated with higher risk perception. Finally, two studies showed an inverse relationship between spirituality/spiritual coping and risk perception [45,46], suggesting a possible relationship between belief systems and interpretation of risk.

## Discussion

In this systematic review of 53 studies of patients at high risk for cancer we identified several key clinical, demographic, and psychosocial factors associated with perceived risk of acquiring cancer in these patient populations. These are depicted in Figure 1. These results highlight known and unknown factors related to the science of risk perception assessment in patients at higher than average risk for cancer.

Most of the studies evaluated in this review used an observational design studying mostly women and their perceived risk of developing breast or ovarian cancer often in the context of genetic testing. In contrast, little literature was found on factors influencing risk perception of acquiring other common cancers such as prostate and colorectal cancer. Clinical factors associated with cancer risk perception included the extent of family history, as well as previous preventive tests and treatments. Demographic factors including age, race/ethnicity, and education level may play a role in risk perception. No studies were designed to assess factors influencing risk perception in a prospective and stratified manner for groups such as ethnic minorities or elderly populations.

Based on this literature, cognitive factors including beliefs about the preventability and severity of the condition, as well as the ability to process numerical information may be important in risk perception. We also observed consistently reported associations between affective factors such as distress, depression, worry and risk perception. These are perhaps the best characterized factors influencing cancer risk perception in high risk patients. A limited number of personality and coping factors may also relate to risk perception.

Our findings complement those of previous systematic reviews by highlighting the strides taken in describing key factors influencing cancer risk perception, especially affective factors. Like Vernon, we focused on key cognitive and affective correlates of cancer risk perception in hopes that a more complete description of such factors could empirically inform future interventions. Unlike Vernon, we focused on groups at high risk, believing the specific needs of these populations are under-explored, increasingly salient, and distinct from the general population. Katapodi found weak but significant associations between perceived risk and age, education, race, and worry; our findings five years later across multiple cancers demonstrate analogous associations. Each of these domains deserves further exploration in longitudinal and experimental studies.

These data suggest several limitations of the current literature on cancer risk perception among those with a potential inherited predisposition to cancer. Taking the next step in improving the measurement of risk perception such as better discriminating between the merits of rating comparative risk versus estimating objective risk could help standardize the cancer risk perception literature. Another limitation to current conceptions of risk relates to current modes of measurement which are generally uni-dimensional (i.e. measuring magnitude or frequency of risk, but not both) and/or contain only single item measures. Multi-dimensional measures that capture frequency, magnitude, as well as individual- and social-level aspects of risk perception need to be developed and utilized. In addition, few studies employed sophisticated measurement or analytic techniques like latent variable modeling, path analysis, or the like. Along with experimental study designs in which investigators would specify *a priori *hypothesized relationships and the direction of those relationships, these techniques would provide a sound basis for causal inferences related to risk perception research to be made.

Finally, this systematic review did not identify potential rich networks of social and behavioral influences that may cause some persons to deeply engage with their risk, while others seem to push it aside. Such dynamics related to the salience of perceived risk information would be important to address in more sophisticated analyses that contextualize risk in the broad fabric of patients' lives and social networks.

## Clinical Implications

Research defining correlates of perceived risk of acquiring cancer among those with elevated cancer susceptibility suggest several clinical implications. Interventions to address perceived risk of developing cancer among high risk populations should not only rely on facts about clinical and demographic characteristics, but also on the real psychosocial factors that may influence risk perception for those at high risk for cancer. Risk perception is not merely a cognitive process, but an affective and existential one. The relationship between worry and risk perception that we so consistently observed suggests that worry may influence screening behaviors among high risk patients regardless of the whether sound evidence exists for the clinical utility of those screening tests, such as in the case of direct-to-consumer genomic testing. This creates tensions for practicing clinicians who strive to judiciously utilize tests in a manner consistent with the best available evidence and the patient's values. Risk perception is an unavoidably affectively-loaded filter and is thus quite susceptible to manipulation and distortion, making approaches to informed decision-making that acknowledge and work in concert with these influences in high risk populations especially challenging and necessary.

## Conclusions

Overall, these data suggest that the science of characterizing and improving risk perception in cancer, although evolving, is still relatively undeveloped at least in several key clinical topic areas. First, future studies focusing on cancer risk perception among men, racially/ethnically diverse populations that experience cancer disparities and the elderly would add considerable value to the literature. Research dedicated to risk perception related to specific topics such as adoption of lifestyle behaviors, the use of complementary/integrative medicine, genomic technologies in prediction and prognostication, or participation in research studies including chemoprevention would have potentially wide-reaching implications for cancer control initiatives.

## Competing interests

The authors declare that they have no competing interests.

## Authors' contributions

JT conceived of the study and, along with PE, MM, designed the study search strategy. JT, KJ, PS, DE, BC, JC, SE, and KN reviewed study abstracts and extracted data from full text articles. ML assisted with acquisition of articles and helped design data abstraction forms. JT, KJ, PS, DE, KN, and MM helped to draft the manuscript. All authors read and approved the final manuscript.

## Supplementary Material

Additional file 1 **Appendix**. Table showing details about risk perception measures used in reviewed studies. Click here for file
