# Supplementary material for: Factors Influencing Cancer Risk Perception in High Risk Populations: A Systematic Review
Source: Hered Cancer Clin Pract. 2011 May 19;9(1):2. doi: 10.1186/1897-4287-9-2 (PMC3118965; doi:10.1186/1897-4287-9-2)
Supplement: Additional file 1 — Appendix. Table showing details about risk perception measures used in reviewed studies. [file 1897-4287-9-2-S1.DOC]

Appendix

|  | **Categorical Measure** | | **Continuous Measure** | | **How many measures used?** | **Was accuracy of risk perception measured?** | **Other details about measures** | **Genetic or Non-Genetic Study?** | **If Genetic: Were Mutation Carriers Included?** |
| --- | --- | --- | --- | --- | --- | --- | --- | --- | --- |
|  | **Comparative Risk** | **Absolute Risk** | **Comparative Risk** | **Absolute Risk** |  |  |  |  |  |
| Domanska, 2007 [29] |  | X |  |  | 1 | Yes | Risks in 20% intervals | Genetic | Yes – HNPCC mutation carriers |
| Haas, 2005 [30] | X |  |  |  | 1 | Yes | Chances of getting BC in lifetime… much lower than avg woman, little lower, same etc. | Non-Genetic |  |
| Rowe, 2005 [31] | X |  |  | X | 2 | No | Scale 0% to 100% likelihood develop BC and stay free from BC for life (separate). Compared to other women your age w/ similar cancer family history your likelihood of developing is much higher, higher, same, etc. | Non-genetic |  |
| Gil, 2003 [47] |  |  |  | X | 1 | Yes | A visual  analogous scale was used by the participants to  assess their perceived risk of breast cancer from 0  to 100% (not at all*-*very much) | Non-genetic |  |
| Lebel, 2003 [34] |  |  |  | X | 1 | Yes | Participants evaluated their personal risk of a positive  biopsy finding on a scale from 1 to 10 | Non-genetic |  |
| Fang, 2003 [48] | X |  |  |  | 1 | No | “Compared to an average  woman your age with a family history of ovarian cancer,  what are your chances of getting ovarian cancer someday?”  Responses to this item ranged from 1, “much less,” to 5,  “much more.” | Non-genetic |  |
| Cappelli, 2001 [9] |  |  |  | X | 1 | No | number of statements  asked participant to estimate her risk of getting various types of cancer (breast, ovarian and any other). placed a checkmark on a linear scale which ranged from 0% (no chance) to 100% (absolutely certain). avg perceived risk variable created by summing responses from the scale items divided by the # of items. | Genetic | *BRCA1/BRCA2 mutations* |
| Hatcher, 2001 [26] |  | unclear |  |  | Multiple measures? | No | “risk perception questionnaire”;  Reported perceived risk levels of between 1 in 2 and 1 in 4, | Genetic |  |
| Wellisch, 2001 [49] |  |  |  | X | 1 | Yes | estimate of their likelihood of developing  BC in their lifetime, on a scale of 0% to 100%,  with higher scores indicating higher perceived risk of developing  BC | Non-genetic |  |
| Audrain, 1997 [35] | X |  |  |  | 1 | No | “In your opinion, compared to other women your age, what are your chances of getting BC/OC? (1=much lower, 5=much higher)” | Non-genetic |  |
| Schwartz, 1995 [43] | X |  |  |  | 1 | No | 3 comparative questions on ovarian cancer on a 4 point likert scale | Non-genetic |  |
| Zikmund-Fisher, 2008 [50] |  | X |  |  | 3 | No | (1) ‘‘taken all together, how worried would you be about getting any of the above health conditions if you did take tamoxifen’’, (2) ‘‘taken all together, how common do you think the above health conditions are for women who take tamoxifen’’, and (3) ‘‘if you were to choose to take tamoxifen, how likely do you think you would be to experience a side effect?’’ Participants responded to each question on a 5-point scale, with 1 representing not at all worried/common/likely and 5 representing extremely worried/common/likely. | Non-genetic |  |
| Mellon, 2008 [3] | X |  |  |  | 1 | No | Multiple comparative questions dealing w/ BC and OC; five-point Likert scale  ranging from ‘much lower’ to ‘much higher’ | Non-genetic |  |
| Peterson, 2008 [56] | X |  |  | X | 2 | No | ‘In your opinion, compared with other persons your age, would you say your chances of getting  cancer are: 1-much lower, 2-a little lower, etc and rate their perceived  risk of having a deleterious p53 gene mutation on a continuous scale ranging from 0 to 100% | Genetic | P53 |
| Codori, 2005 [33] |  |  |  | X | 1 | Yes | Visual analog scale consisting of a line with the anchors,  “*definitely will not get colon cancer*” and “*definitely will get colon cancer*.” Participants marked the line indicating their attitude, and the distance between the  mark and the anchors was converted to percent risk. | Genetic | hMSH2, hMLH1,  hMSH6, hPMS1, and hPMS2 |
| Claes, 2004 [57] |  | X |  |  |  | Yes | Participants had to recall the numerical  risk (a percentage) of being a carrier of a mutation. Subjective risk perception was measured by a verbal scale ranging from “I am convinced that I am not a carrier of a  mutation (=1)” to “I am convinced that I am a carrier of mutation (=5)” with the midpoint (3) “my risk of being a carrier is as high as my risk of not being a carrier” | Genetic | hMLH1 and hMSH2 |
| Bruno, 2004 [58] | X |  |  |  | 1 | No | personal risk for developing breast cancer during their lifetime compared with women of the same age. Possible responses were ‘lower’ ‘similar’ or ‘higher | Genetic | BRCA1/2 mutation |
| Salsman, 2004 [13] |  |  | X | X | 2 | Yes | ‘‘What are the chances you will  develop OC some day?’ and ‘‘What are the chances that the  average woman your age will develop OC some day?’’ 0-100% | Non-genetic |  |
| Beebe-Dimmer, 2004 [40] |  |  |  | X | 1 | No | “On a scale of 0 to 10, where 0 is certain not to happen and 10 is certain to happen, how likely are you to get prostate carcinoma in the next 10 years / in your lifetime?” | Non-genetic |  |
| Lobb, 2004 [22] |  | X |  |  | 1 | Yes | a potentially high-risk category (25–80% lifetime risk of BC), a moderate-risk category (12–25%) and an  avg-risk category (9–12%) | Non-genetic |  |
| Van Dijk, 2003 [18] | X |  |  | X | 2 | Yes | Compared to the avg Dutch woman, my risk of developing BC (again) is… (1-7)  My risk of developing BC (again) is _ out of 100 | Genetic | BRCA1 and BRCA2 |
| Hensley, 2003 [59] | X |  |  | X | 2 | No | Perception of lifetime risk of developing  OC was determined by a query [16] asking each participant to state whether she perceived her risk, “compared with other women my age” and “compared with other women with similar family history” to be “much more,” “slightly more,” “about the same,” “slightly less,” or “much less.” In addition, each woman was asked to provide, on a scale from 0 to 100%, her perceived lifetime risk of OC. | Genetic | *BRCA1* or *BRCA2*  mutation |
| Andrykowski, 2002 [36] |  |  | X | X | 2 | No | What are the chances that you will develop BC some day? And “What are the chances that the avg woman your age will develop BC someday?” 0-100% | Non-genetic |  |
| Royak-Schaler, 2002 [32] | X | X |  |  | 2 | No | how likely she thought it was that she might develop BC in her lifetime. 4-point Likert scale: "very unlikely," "somewhat unlikely", etc. assess what she thought her chances were of developing BC someday, compared with most women her age. 5-point Likert scale, ranging  from "much lower" to "much higher." | Non-genetic |  |
| Di Prospero, 2001 [24] |  | X |  |  | 1 | No | 0% to 100%, using increments of 10% | Genetic | *BRCA1* and *BRCA2*  mutations |
| Elit, 2001 [25] | X | X |  | X | 3 | Yes | Various questions on OC risk | Non-genetic |  |
| Vernon, 2001 [27] |  | X |  |  | 1 | No | four response categories from strongly agree (4) to strongly disagree (1). I believe that the chance I might develop colorectal cancer is high; I think it is very likely that I will develop colorectal cancer or polyps; and I believe that the chance that I will develop colorectal polyps is high. | Non-genetic |  |
| Collins, 2000 [39] |  | X |  | X | 2 | No | risk of developing bowel cancer as compared with the general population. graded scale: much higher; higher; slightly higher; the same; slightly lower; lower; much lower; and don’t know. lifetime risk of developing bowel cancer on a visual analogue scale  (VAS) from 0 ‘no chance at all’ to 100 ‘absolutely certain’ | Non-genetic |  |
| Erblich, 2000 [38] |  |  |  | X | 1 | No | How likely they felt they were to develop BC sometime during their lives. 0%(not at all likely) to 100% (extremely likely) | Non-genetic |  |
| Bratt, 2000 [41] |  | X |  |  | 1 | Yes | general and personal lifetime risk of prostate cancer illness. 1 in 100 (1%); 1 in 20 (5%); 1 in 10 (10%); 1 in 5 (20%); 1 in 3 (33%); 1 in 2 (50%); 2 in 3 (67%); 4 in 5 (80%); and almost certain  (close to 100%). | Genetic | Subjects had a 50% likelihood of having a mutation in a dominantly inherited prostate cancer susceptibility gene. |
| Glanz, 1999 [16] | X |  |  |  | 1 | No | how high the respondents believed their chances of getting colon cancer or polyps were, compared with other people their age. (1 - much lower to 5- much higher) | Non-genetic |  |
| Codori, 1999 [17] |  |  |  | X | 1 | No | evaluated on a scale from 0 (definitely  will not get colon cancer) to 100 (definitely will get colon cancer) | Genetic | HNPCC mutations |
| Zakowski, 1997 [19] |  |  |  | X | 1 | Yes | Likelihood of developing BC in their lifetime from 0 (not at all likely) to 100 (extremely likely) | Non-genetic |  |
| Stefanek, 1995 [51] |  |  |  | X | 1 | Yes | Estimate her % likelihood (0-100%) of developing BC over the 10 and 30 year periods | Non-genetic |  |
| Lerman, 1994 [28] |  |  |  | X | 1 | No | Perceived relative risk. 5 pt Likert scale. Or perceived likelihood of developing BC | Non-genetic |  |
| Rimes, 2006 [15] |  |  |  | X | 1 | Yes | likelihood of developing cancer, participants were asked to report their “feelings rather than what [they thought] a ‘rational’ or ‘correct’ response would be.” 0-100 | Genetic | a dominantly inherited cancer predisposing  gene |
| Bondy, 1992 [52] |  | X |  |  | 1 | Yes | perceived lifetime risk of breast cancer (small, moderate, high) | Non-genetic |  |
| Blalock, 1990 [14] | X | X |  |  | 2 | Yes | How likely they were to get CRC sometime in their life and then to others their age. 5 pt scale (very unlikely/a lot lower to very likely/a lot higher.) | Non-genetic |  |
| Schwartz, 2000 [46] | X |  |  |  | 1 | No | Likert-style item (19): “In  your opinion, compared to other women your age, what are your chances of developing breast/ovarian cancer again?” (1 = much lower to 5 = much higher). | Genetic | BRCA1/2 |
| Watson, 1999 [23] | X |  |  | X | 2 | Yes | own *lifetime* chances of breast cancer based on the family history (expressed as a 1 in x odds ratio), *relative* risk (chances of developing breast cancer compared with the average woman, on a 5-point scale, from ‘very much lower’ to ‘very much higher than average’ | Non-genetic |  |
| Cunningham, 1998 [37] | X |  |  |  | 1 | Yes | 2 questions to create comparative. 1=no chance, 2 = 1 in 100, 3= 1 in 50, etc | Non-genetic |  |
| Miller, 2005 [42] | X |  |  |  | 1 | Yes | estimate their perceived risk for breast cancer by comparing their personal chances of getting breast cancer with women their same age, using a scale of 1  (i.e., very much lower than avg) to 5 (i.e., much higher than avg) | Non-genetic |  |
| Emery, 2007 [21] | X |  |  |  | 1 | Yes | Risk perception was measured on a scale of 1–7, relative to the general population, considering 1 as  ‘much less likely’ and 7 as ‘much more likely to develop breast/bowel cancer than other people of your age’ | Non-genetic |  |
| Bjorvatn, 2007 [20] | X | X |  | X | 3 | Yes | perceived risk of  developing cancer as a percentage (0–100%). Secondly,  patients rated their own risk of cancer in  words, using a six-point category rating scale (unlikely= 1, no doubt=6). Compared their own risk to that of other persons of the same  age and gender (peers), on a five-point scale | Non-genetic |  |
| van Oostrom, 2007 [60] |  | X |  |  | 1 | No | ‘‘Independent of my actual risk, I feel my risk of developing  cancer is ‘not likely’ (1) to ‘very likely’’’ (7). | Genetic | BRCA1/2 mutation |
| Quillin, 2006 [45] | X | X |  |  | 4 | No | risk for breast cancer | Non-genetic |  |
| O’Neill, 2006 [44] |  |  |  | X | 1 | No | how certain they were that they would be diagnosed, or diagnosed again, with breast cancer on a scale from 0 to 100 | Genetic | *BRCA1/2* mutation |
| Matloff, 2006 [12] |  |  | X |  | 1 | No | Avged comparative breast cancer development. 0% (not at all likely)  to 100% (extremely likely) | Genetic | BRCA1 or  BRCA2 mutation |
| Lipkus, 2006 [53] | X | X |  |  | 2 | No | chance of getting CRC in their lifetime. | Non-genetic |  |
| Martin, 2006 [11] |  | X |  |  | 1 | No | general likelihood  of getting breast cancer, chances of getting breast cancer in the next few years, and chances of getting breast cancer  ‘‘sometime during my life.’’ 5 pt likert | Genetic | BRCA1/2 excluded |
| Cameron, 2006 [54] |  | X |  |  | 1 | No | ‘‘How likely do you think it is that, at some point in your life, you will get breast cancer?’’ (0-not at all to 6-almost certain) | Non-genetic |  |
| Madalinska, 2005 [55] |  |  |  | X | 1 | No | rate their self-perceived risk on a scale 0% to 100%, where 0 corresponded to no risk at all and 100  corresponded to being certain about developing cancer in the future. | Non-genetic |  |
| Cappelli, 2005 [10] | X |  |  |  | 1 | No | Used Breast Cancer Survey; Perceived risk of getting BC compared with other teens (lower, same little higher, much higher); perceived risk of carrying BRCA gene mutation (not at all, little, somewhat, very likely) | Non-genetic |  |
